# The Relationship of Grade, Stage and Tobacco Usage in Head and Neck Squamous Cell Carcinoma With p53, PIK3CA and MicroRNA Profiles

**DOI:** 10.7759/cureus.54737

**Published:** 2024-02-23

**Authors:** Kamini Kiran, Nilotpal Chowdhury, Ashok Singh, Manu Malhotra, Sanjeev Kishore

**Affiliations:** 1 Oral Pathology, All India Institute of Medical Sciences, Rishikesh, Rishikesh, IND; 2 Pathology and Laboratory Medicine, All India Institute of Medical Sciences, Rishikesh, Rishikesh, IND; 3 Pathology/Histopathology/Renal Pathology, All India Institute of Medical Sciences, Rishikesh, Rishikesh, IND; 4 Otorhinolaryngology and Head-Neck Surgery, All India Institute of Medical Sciences, Rishikesh, Rishikesh, IND

**Keywords:** tnm staging, molecular profiling, microrna (mirna), immunohistochemical markers, hpv infection, head and neck squamous cell carcinoma

## Abstract

Background: Head and neck squamous cell carcinoma (HNSCC) has multiple epigenetic modifications including post-transcriptional regulation by microRNAs (miRNAs) as well as alterations in molecular pathways due to mutations. Examining these miRNAs and location-specific molecular alterations is essential to understanding the intricacies of HNSCC and directing focused diagnoses and treatments.

Aim: To investigate tobacco-related changes in the expression of miRNAs and proteins with clinicopathological parameters of HNSCC and disease-modifying personal habits like tobacco and alcohol use.

Methodology: The study concentrated on oropharyngeal cancers using immunohistochemistry and reverse transcription-polymerase chain reaction. Expression of microRNAs mir15a, mir20b, mir21, mir31, mir33b, mir146a, mir155, mir218, mir363 and mir497 and immunohistochemical expression of P53 and PIK3CA were correlated with grade, stage and personal habits like tobacco and alcohol intake.

Results: mir21 and mir15a are under-expressed in higher grades with a trend towards statistical significance (P-value of 0.094 and 0.056 by one-way analysis of variance (ANOVA) on ΔC_T _values). mir155 and mir146a are overexpressed in stage IV tumours while mir 31 is under-expressed in stage IV tumours but statistical significance was not reached. mir497 showed overexpression in tobacco users, but these results were limited by many tumours not showing any amplification for the miRNA and statistical significance was not reached. There was no statistically significant association found between immunohistochemical expression of p53 and PIK3CA with grade, stage or personal habits.

Conclusion: Through the deciphering of complex miRNA patterns and their relationships with clinicopathology, this study attempted to increase our understanding of HNSCC. Some candidate miRNAs showing probable association with grade, stage and personal habits were identified, but larger studies are needed to confirm or refute the importance of these miRNAs.

## Introduction

Cancers affecting the mucosal lining of the mouth, throat, and larynx are collectively known as head and neck squamous cell carcinomas (HNSCCs). The complex interaction between viral, environmental, and genetic variables leads to the multifactorial aetiology of HNSCC [1]. Human papillomavirus (HPV)-associated HNSCC, especially in the oropharyngeal area, has demonstrated unique clinical and molecular characteristics [2,3]. Tobacco and HPV work synergistically to promote carcinogenicity [4].

The literature provides extensive information on HPV-related HNSCC, highlighting debates, novel ideas, and cutting-edge treatments. Nonetheless, there is a pressing need to investigate the regional variations of HNSCC and the effects of tobacco use and HPV infection on molecular changes in various head and neck anatomical locations [5-7].

Impact of microRNAs (miRNAs) and proteins

Proteins and miRNAs are key components of the pathophysiology of HNSCC. Deciphering their changes in a location-specific way is essential to understanding the complexity of the illness. To shed light on the molecular pathways, diagnostics, and therapy options using nanomedicine, Bhattacharjee et al. [3] investigated the pharmacological influence of miRNAs in HNSCC. With respect to the molecular and clinical implications, Powell et al. (2021) highlighted the fundamental distinctions between head and neck malignancies that are HPV-positive and HPV-negative [8].

Even though there is an extensive body of literature on HNSCC, there remain unanswered questions, especially regarding region-specific molecular changes. Sabatini et al. discussed the intricate mechanisms underlying viral-associated carcinogenesis in head and neck cancers. The regulatory roles and possible implications of miRNAs for targeted therapeutics were highlighted in an investigation of the miRNAs landscape in head and neck cancer [5].

Immunohistochemical (IHC) markers (P53, PIK3CA)

In HNSCC, understanding the molecular pathways and predicting patient outcomes is greatly aided by the use of various IHC markers, especially in the context of HPV infection. Salazar et al. (2014) and Smith et al. (2010) have identified p53 as important markers associated with survival in HNSCC [9,10]. Aguayo et al. (2023) and Kommineni et al. (2015) studied the significance of PIK3CA mutations in the PI3K/AKT/mTOR signalling pathway [11,12]. The role of PIK3CA in HPV-positive oropharyngeal squamous cell carcinoma was studied by Chiosea et al. (2013) [13]. These IHC markers collectively offer valuable information on the molecular profile of HNSCC.

To fill research gaps, the present study examined grade, stage and tobacco-related changes in proteins and miRNAs in HNSCC.

## Materials and methods

The present study was conducted in the Department of Pathology and Lab Medicine, at the All India Institute of Medical Sciences, Rishikesh, India. The study was approved by the institution’s ethical committee (IEC - AIIMS/IEC/18/505). This was an exploratory study having a sample size of one hundred and fifty HNSCC samples.

Histologically confirmed cases of malignant squamous lesions of the oropharyngeal tracts, which include the tongue, buccal mucosa, floor of the mouth, alveolus, maxilla, and larynx, were retrieved from archival blocks and included and patients with a history of previous radiation therapy were excluded from the study.

Archived formalin-fixed paraffin-embedded (FFPE) tissue sections (3 μm) were carefully deparaffinized, rehydrated, and pretreated with antigen retrieval. Using antibodies that target important indicators, an IHC examination was performed.

**Figure 1:**
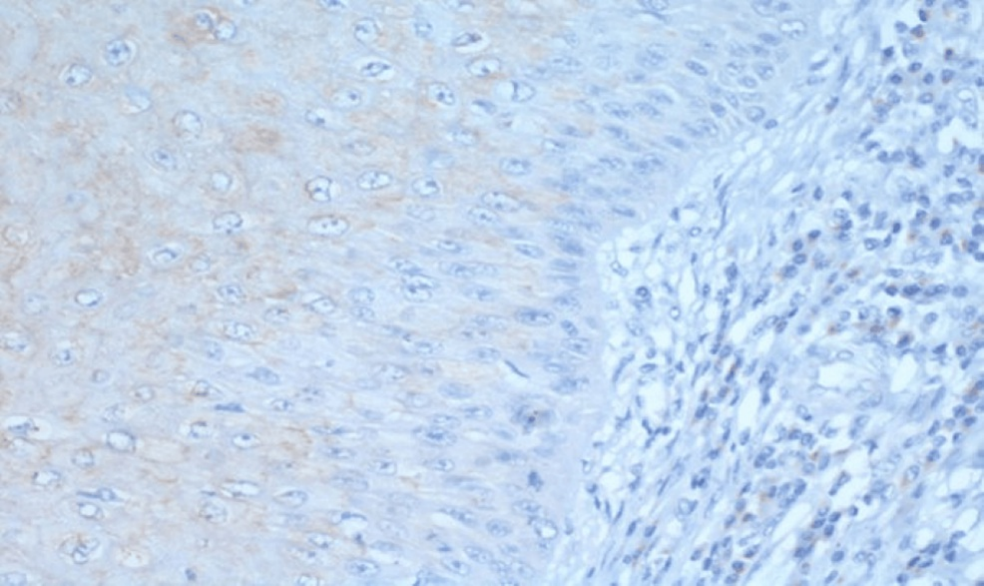
The figure represents a photomicrograph of IHC PIK3CA (magnification 40x) IHC - immunohistochemistry

**Figure 2:**
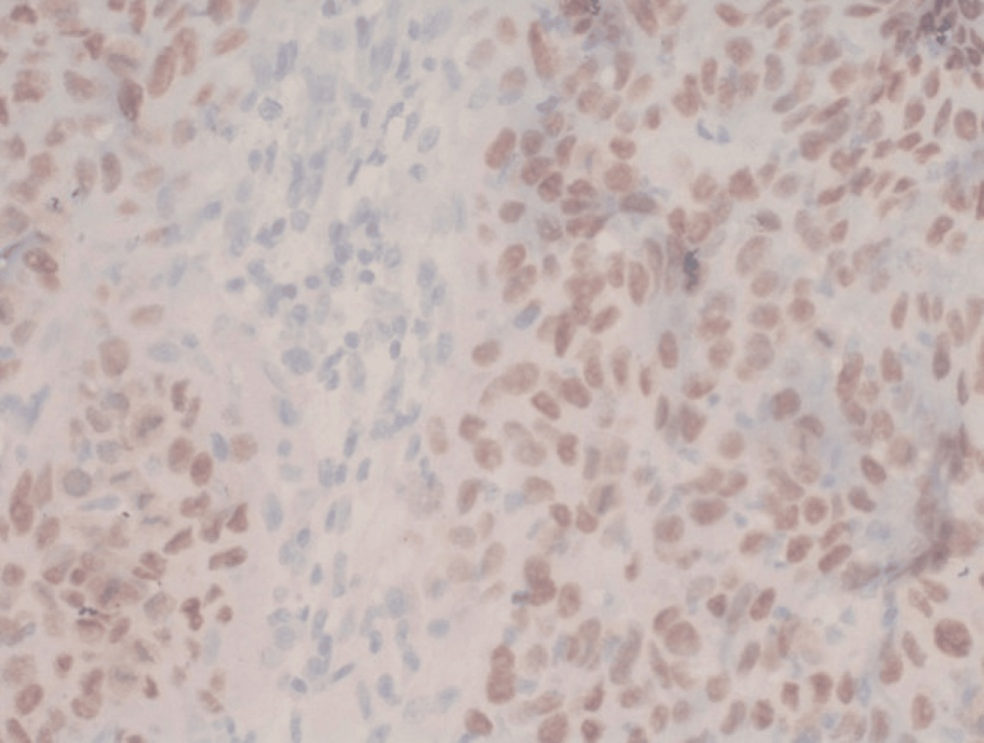
The figure represents a photomicrograph of IHC p53 (magnification, 40x) IHC - immunohistochemistry

Antibodies against PIK3CA and P53 (DO7) (Invitrogen, Thermofisher, USA) were used in the IHC analysis. Based on predetermined scoring criteria for PIK3CA and P53, the assessment of positive and negative staining for each marker involved a minimum evaluation of 10 high-power fields of invasive cancer. In PIK3CA both cytoplasmic and nuclear positivity were taken positive and in p53 nuclear positive staining was taken as positive. Representative pictures of IHC are given in Figures 1-2.

The mirVana miRNA Isolation Kit (Invitrogen, Thermofisher, USA) was used to isolate RNA from FFPE tissue sections. The following miRNA were studied: mir15a, mir20b, mir21, mir31, mir33b, mir146a, mir155, mir218, mir363 and mir497. The concentration and purity of RNA were estimated using a Tecan reader (infinite F200 PRO, Germany). Synthesis of cDNA was carried out using the Mir-X miRNA First-Strand Synthesis Kit (Takara Bio, USA). U6 was taken as a housekeeping gene. Reverse transcription-polymerase chain reaction (RT-PCR) was carried out using a Bio-Rad CFX96 machine (Bio-Rad Laboratories, Hercules, CA). The cycle threshold (Ct) for each miRNA was estimated from the inbuilt software of the instrument. Delta Ct (ΔCt) values for each miRNA were estimated by subtracting the mean Ct of the miRNA probe from the mean Ct of the housekeeping U6 gene. ΔΔCt values were then calculated by subtracting the ΔCt values of the subgroup of interest from the ΔCt of a reference subgroup. For the relative expression between different grades, well-differentiated carcinoma formed the reference subgroup, against which the relative expression of moderately differentiated and poorly differentiated carcinomas was studied. For stage, stage I formed the reference subgroup, against which the relative expression of Stage II, Stage III, Stage IVA, and Stage IVB were studied. For personal habits, patients not having either tobacco or alcohol use was the reference subgroup, against which the relative expressions of the subgroups showing tobacco use alone or tobacco and alcohol use were studied. Only one subject reported alcohol use alone for whom relative expression was not calculated. Relative miRNA expression levels were calculated using the 2^-ΔΔCt^ method, normalized to the housekeeping gene U6. Statistical significance was tested using one-way analysis of variance (ANOVA) on ΔCt values.

## Results

This paper provides an examination of HNSCC, emphasizing the role of tobacco use, grade and stage with changes in p53 and PIK3CA protein and miRNA expression. mir21, mir146a, mir155 and mir31 showed amplification in more than 80% of the tumours (139/150 for mir21,133/150 for mir146a,131/150 for mir155 and 122/150 for mir31). mir15a showed amplification in around 75% (112/150 tumours). mir20b, mir33b, mir218, mir363 and mir497 showed no amplification in more than one-fourth of the tumours and hence the interpretability of these mirs was limited.

miRNA expression with grade

Table 1 displays the differences in miRNA expression between the different grades of HNSCC. Notably, mir21 and mir15a are underexpressed in higher grades with a ΔΔCT of more than 2 and a trend towards statistical significance. Though mir33b and mir 497 also showed large-fold changes, the results for these miRNAs are limited by a large number of tumours showing no expression of that particular miRNA.

**Table 1:** Relationship of miRNA ΔCt values with the grade of head and neck squamous cell carcinoma. The data is represented by mean ±SD and the number of cases showing expression and no expression of the microRNA. The relative expression or fold change is estimated by the ΔΔCt method. The P-value is estimated by the one-way analysis of variance (ANOVA) test of the cases which showed miRNA expression. A P-value below 0.05 was taken as significant and a P-value between 0.05 and 0.1 was interpreted as a trend towards significance.

miRNA	Grade	Mean±SD of ΔCt	Number of cases showing expression	Number of cases showing no expression of miRNA	ΔΔCT	Relative expression (2^-^^ΔΔCT^)	P value
mir155	Well-differentiated	5.98±4.16	54	13	NA	NA	0.33
	Moderately differentiated	6.04±4.02	69	5	0.06	0.96	
	Poorly differentiated	8.25±4.72	8	1	2.27	0.21	
mir146a	Well-differentiated	6.07±3.48	56	11	NA	NA	0.51
	Moderately differentiated	6.54±4.03	68	6	0.47	0.72	
	Poorly differentiated	7.63±5.69	9	0	1.56	0.34	
mir218	Well-differentiated	9.04±5.30	41	26	NA	NA	0.87
	Moderately differentiated	9.05±3.99	43	31	0.01	0.99	
	Poorly differentiated	7.60±9.16	3	6	-1.44	2.71	
mir31	Well-differentiated	7.69±4.29	53	14	NA	NA	0.156
	Moderately differentiated	9.07±3.14	62	12	1.39	0.38	
	Poorly differentiated	8.98±6.37	7	2	1.29	0.41	
mir21	Well-differentiated	1.13±4.68	61	6	NA	NA	0.094
	Moderately differentiated	2.74±5.68	69	5	1.62	0.33	
	Poorly differentiated	4.37±5.45	9	0	3.24	0.11	
mir497	Well-differentiated	8.54±4.07	40	27	NA	NA	0.097
	Moderately differentiated	10.56±3.61	39	35	2.02	0.25	
	Poorly differentiated	10.92±10.36	4	5	2.38	0.19	
mir363	Well-differentiated	9.78±4.09	33	34	NA	NA	0.70
	Moderately differentiated	10.56±3.91	41	33	0.78	0.58	
	Poorly differentiated	10.34±3.05	3	6	0.56	0.68	
mir33b	Well-differentiated	8.72±5.31	42	25	NA	NA	0.19
	Moderately differentiated	9.48±4.46	53	21	0.76	0.59	
	Poorly differentiated	12.13±4.76	8	1	3.41	0.09	
mir20b	Well-differentiated	8.84±6.38	18	49	NA	NA	0.90
	Moderately differentiated	8.48±5.25	14	60	-0.36	1.28	
	Poorly differentiated	6.20 ±NA	1	8	-2.65	6.27	
mir15a	Well-differentiated	7.02±4.16	51	16	NA	NA	0.05
	Moderately differentiated	8.99±3.96	55	19	1.96	0.26	
	Poorly differentiated	9.53±9.02	6	3	2.51	0.18	

miRNA expression with stage

Table 2 shows the changes in the expression of miRNA between the HNSCC stages (I-VB). In moderately differentiated squamous cell carcinoma (MDSCC), mir155 and mir146a are overexpressed in stage IV tumours while mir31 is underexpressed in stage IV tumours. However, statistical significance was not achieved at any stage.

**Table 2:** Relationship of miRNA ΔCt values with stage of head and neck squamous cell carcinoma. The data is represented by mean ±SD and the number of cases showing expression and no expression of the microRNA. The relative expression or fold change is estimated by the ΔΔCt method. The P-value is estimated by a one-way analysis of variance (ANOVA) test of the cases which showed miRNA expression. A P-value below 0.05 was taken as significant and a P-value between 0.05 and 0.1 was interpreted as a trend towards significance.

miRNA	Stage	Mean±SD of ΔCt	Number of cases showing expression	Number of cases showing no expression of miRNA	ΔΔCt	Relative expression (2^-^^ΔΔCt^)	P value
mir155	I	6.68±3.94	11	0	NA	NA	0.33
	II	6.20±4.25	16	2	-0.48	1.40	
	III	6.15±3.39	21	5	-0.53	1.44	
	IVA	6.44±4.42	74	11	-0.24	1.18	
	IVB	3.07±2.12	9	1	-3.62	12.26	
mir146a	I	7.15±4.28	11	0	NA	NA	0.51
	II	5.95±5.87	17	1	-1.19	2.28	
	III	6.61±2.53	20	6	-0.53	1.45	
	IVA	6.61±3.82	76	9	-0.53	1.45	
	IVB	4.33±2.01	9	1	-2.81	7.02	
mir218	I	8.77±2.32	7	4	NA	NA	0.99
	II	9.29±3.17	6	12	0.52	0.70	
	III	8.53±5.21	20	6	-0.24	1.18	
	IVA	9.18±5.44	45	40	0.41	0.75	
	IVB	9.09±2.77	9	1	0.32	0.80	
mir31	I	6.16±3.34	10	1	NA	NA	0.24
	II	8.84±4.59	16	2	2.68	0.16	
	III	7.65±3.34	20	6	1.49	0.36	
	IVA	8.88±4.12	67	18	2.72	0.15	
	IVB	9.09±1.73	9	1	2.93	0.13	
mir21	I	1.07±6.16	11	0			0.93
	II	2.89±6.73	16	2	1.83	0.28	
	III	1.92±4.90	22	4	0.85	0.55	
	IVA	2.23±5.10	80	5	1.16	0.45	
	IVB	1.84±5.09	10	0	0.77	0.58	
mir497	I	8.58±1.83	7	4	NA	NA	0.62
	II	9.81±4.64	8	10	1.23	0.43	
	III	8.34±4.73	16	10	-0.25	1.19	
	IVA	9.97±4.74	44	41	1.39	0.38	
	IVB	10.8±12.04	8	2	2.22	0.21	
mir363	I	11.65±2.28	6	5	NA	NA	0.56
	II	9.43±2.78	7	11	-2.21	4.64	
	III	9.16±4.41	15	11	-2.49	5.62	
	IVA	10.6±84.35	41	44	-0.97	1.96	
	IVB	9.49±1.99	8	2	-2.16	4.46	
mir33b	I	11.79±4.54	8	3	NA	NA	0.33
	II	9.20±4.11	13	5	-2.59	6.03	
	III	8.37±4.44	16	10	-3.42	10.73	
	IVA	9.66±5.38	58	27	-2.13	4.37	
	IVB	7.13±2.02	8	2	-4.66	25.33	
mir20b	I	12.02±1.71	5	6	NA	NA	0.44
	II	8.04±3.24	3	15	-3.98	15.81	
	III	5.20±7.10	5	21	-6.82	113.18	
	IVA	8.54±6.21	19	66	-3.48	11.18	
	IVB	11.78 ±NA	1	9	-0.24	1.18	
mir15a	I	7.34±4.71	10	1	NA	NA	0.66
	II	8.51±6.62	10	8	1.17	0.44	
	III	7.05±2.95	19	7	-0.29	1.22	
	IVA	8.32±4.66	65	20	0.98	0.51	
	IVB	9.57±2.62	8	2	2.23	0.21	

miRNA expression by associated habit

In Table 3, the relationship of miRNA expression with lifestyle choices (alcohol, tobacco, alcohol and tobacco) is given. Only mir497 showed overexpression in tobacco users, but these results were limited by many tumours not showing any amplification for the miRNA.

**Table 3:** Relationship of miRNA ΔCt values with associated habits of patients suffering from head and neck squamous cell carcinoma. The data is represented by mean ±SD and the number of cases showing expression and no expression of the microRNA. The relative expression or fold change is estimated by the ΔΔCt method. The P-value is estimated by a one-way analysis of variance (ANOVA) test of the cases which showed miRNA expression. A P-value below 0.05 was taken as significant and a P-value between 0.05 and 0.1 was interpreted as a trend towards significance.

miRNA	Habit	Mean±SD of ΔCt	Number of cases showing expression	Number of cases showing no expression of miRNA	ΔΔCt	Relative expression (2^-^^ΔΔCt^)	P value
miRNA155	Alcohol	2.27± NA	1	0	NA	NA	0.90
	Nil	6.43± 3.91	24	1	NA	NA	
	Tobacco	6.06± 4.17	79	15	-0.37	1.30	
	Tobacco and Alcohol	6.33± 4.32	27	3	-0.10	1.07	
mir146a	Alcohol	3.91± NA	1	0	NA	NA	0.40
	Nil	6.74± 2.73	24	1	NA	NA	
	Tobacco	6.64± 3.60	82	12	-0.10	1.07	
	Tobacco and Alcohol	5.51± 5.58	26	4	-1.24	2.36	
mir218	Alcohol	10.67± NA	1	0	NA	NA	0.50
	Nil	10.23± 4.55	15	10	NA	NA	
	Tobacco	8.58± 4.60	53	41	-1.65	3.15	
	Tobacco and Alcohol	9.10± 5.64	18	12	-1.13	2.18	
mir31	Alcohol	11.67± NA	1	0	NA	NA	0.70
	Nil	9.08± 3.08	21	4	NA	NA	
	Tobacco	8.34± 3.84	75	19	-0.74	1.68	
	Tobacco and Alcohol	8.20± 4.80	25	5	-0.88	1.84	
mir21	Alcohol	6.90± NA	1	0	NA	NA	0.92
	Nil	2.20± 4.78	24	1	NA	NA	
	Tobacco	2.20± 4.96	85	9	-0.01	1.00	
	Tobacco and Alcohol	1.76± 6.70	29	1	-0.44	1.36	
mir497	Alcohol	10.92± NA	1	0	NA	NA	0.20
	Nil	11.41± 3.87	14	11	NA	NA	
	Tobacco	9.37± 4.43	53	41	-2.04	4.11	
	Tobacco and Alcohol	8.68± 4.42	15	15	-2.73	6.62	
mir363	Alcohol	NaN	0	1	NA	NA	0.36
	Nil	11.14± 3.70	15	10	NA	NA	
	Tobacco	10.26± 3.84	49	45	-0.89	1.85	
	Tobacco and Alcohol	9.03± 4.52	13	17	-2.12	4.33	
mir33b	Alcohol	7.29	1	0	NA	NA	0.77
	Nil	9.94± 4.29	17	8	NA	NA	
	Tobacco	9.12± 4.69	63	31	-0.82	1.77	
	Tobacco and Alcohol	9.75± 6.00	22	8	-0.19	1.14	
mir20b	Alcohol	NaN	0	1	NA	NA	0.95
	Nil	9.09± 6.28	5	20	NA	NA	
	Tobacco	8.36± 5.72	21	73	-0.72	1.65	
	Tobacco and Alcohol	9.01± 6.33	7	23	-0.07	1.05	
mir15a	Alcohol	11.79± NA	1	0	NA	NA	0.40
	Nil	8.84± 3.84	19	6	NA	NA	
	Tobacco	8.21± 4.36	72	22	-0.63	1.55	
	Tobacco and Alcohol	6.96± 5.46	20	10	-1.88	3.68	

IHC marker expression

There was no statistically significant relationship between PIK3CA or p53 with either grade or stage or associated habits (Tables 4-6).

**Table 4:** PIK3CA and P53 IHC expression correlated to associated habits in the study population. Data are represented as counts (N). IHC - immunohistochemistry

IHC marker	Expression	Nil (N)	Tobacco (N)	Tobacco & Alcohol (N)	P value
PIK3CA	Negative	19	78	20	0.157
	Positive	6	16	10	
P53	Negative	9	42	10	0.504
	Positive	15	52	20	

**Table 5:** PIK3CA and P53 IHC expression correlated to TNM stage of HNSCC in the study population. Data are represented as counts (N).

		TNM Staging					
IHC marker	Expression	I (N)	II (N)	III (N)	IVA (N)	IVB (N)	P value
PIK3CA	Negative	9	14	19	68	8	0.956
	Positive	2	4	7	17	2	
P53	Negative	5	7	9	36	5	0.911
	Positive	6	11	17	48	5	

**Table 6:** PIK3CA and P53 IHC expression correlated to the grade of HNSCC in the study population. Data are represented as counts (N). IHC - immunohistochemistry

		Grading			
IHC marker	Expression	Well-Differentiated (N)	Mod differentiated (N)	Poorly differentiated (N)	P value
PIK3CA	Negative	52	60	6	0.585
	Positive	15	14	3	
P53	Negative	23	35	4	0.260
	Positive	44	38	5	

Demographic characteristics

The study included 129 male participants and 21 female participants, the median age was 46 years, ranging from 20 to 82 years and the distribution of the site of the tumours is given in Table 7.

**Table 7:** Distribution of anatomical sites in the study population presented as frequency counts (N) and percentage (%)

Site	Frequency	Percentage
Buccal mucosa	55	36.7%
Tongue	48	32.0%
Alveolus	9	6.0%
Gingivobuccal sulcus	5	3.3%
Maxillary sinus	5	3.3%
Supraglottis	8	5.3%
Hard palate	6	4.0%
Lip	3	2.0%
Mandible	2	1.3%
Other/Multiple	9	6.0%
Total	150	100

## Discussion

The molecular complexities of HNSCC must be fully understood to improve diagnostic and treatment strategies. HNSCC is a significant global health concern. This study focused on miRNAs, which are important molecules in cancer biology because they affect critical cellular functions. The goal of the project was to identify patterns of miRNA expression in various HNSCC subtypes and investigate how these patterns are associated with lifestyle decisions, clinicopathological variables, and the expression of important IHC markers.

miRNAs are important players in the complex dynamics of HNSCC, affecting many aspects including gene expression, grading, and staging. Some patterns stand out, such as the low expression of mir21, mir15a and mir497 in poorly differentiated squamous cell carcinoma (PDSCC), which is consistent with the prognostic relevance observed by Dioguardi et al. (2022) [14]. These findings suggest the possible use of mir21 and mir33b as biomarkers for the severity and course of illness, but need larger studies due to the borderline statistical significance.

Our examination of miRNA expression in relation to TNM staging revealed dynamic patterns indicating possible roles in the advancement of HNSCC. The observed decrease in the expression of mir155 in MDSCC from stage I to stage IVB is consistent with data from Dioguardi et al. (2022) highlighting the significance of using miRNA profiles for accurate staging [14]. Inter-stage differences in the expression of mir146a and mir363 suggest their involvement in distinct stages of HNSCC, which is consistent with the complex molecular dynamics highlighted in earlier studies [4,14-16].

The associations between lifestyle decisions and miRNA expression highlight how the environment potentially affects the biology of HNSCCs. We could not confirm changes in expression of mir155 in tobacco users which was found in Bhat et al. (2018) [17]. We however observed an upregulation of mir497 in alcohol and tobacco users [17,18].

The interaction of genetic and environmental variables in HNSCC was demonstrated by the examination of PIK3CA and P53 IHC markers. We did not find the increased expression of PIK3CA in tobacco smokers which was found in a previous study (Hashmi et al., 2018). No association between lifestyle choices and P53 expression was observed [19].

Understanding the clinical importance of IHC markers is nuanced when viewed in the context of TNM staging and tumour grading. The limited associations observed between IHC markers and HNSCC are consistent with the research conducted by Hashmi et al. (2018) [19]. The function of miRNAs, HPV association, and genetic variation have been explored in several HNSCC-related studies, Sais et al. (2018), Momi et al. (2014), Miller et al. (2015), and Wilkins et al. (2018) [20-23].

To better understand particular indicators and pathways connected to the disease, we examined the findings of the present study in the context of the existing literature on HNSCC and tobacco use. Our findings are consistent with an earlier examination of miRNA dynamics in oral and oropharyngeal squamous cell carcinomas associated with HPV, which identified common patterns of dysregulation, such as the downregulation of mir497 in well-differentiated squamous cell carcinoma (WDSCC) (Salazar et al., 2014) [9].

## Conclusions

The present study has demonstrated complex molecular dynamics in HNSCC, highlighting the crucial function of miRNAs and their interaction with IHC markers and lifestyle decisions. The expression of miRNAs, particularly those associated with disease severity (mir21 and mir33b), provides important information for precise disease classification among HNSCC subtypes.

Understanding the complex interplay among genetics, lifestyle, and molecular pathways in HNSCC is promising in terms of improving the accuracy of diagnosis and customizing treatment approaches. However, larger studies are required since statistical significance was not reached. It is essential to carry out such studies, both now and in the future, to improve patient outcomes and influence the management of HNSCC.
